# Infants Produce Optimally Informative Points to Satisfy the Epistemic Needs of Their Communicative Partner

**DOI:** 10.1162/opmi_a_00166

**Published:** 2024-10-04

**Authors:** Tibor Tauzin, Josep Call, György Gergely

**Affiliations:** Department of Cognitive Science, Central European University; Institute of Linguistics, University of Vienna; School of Psychology and Neuroscience, University of St Andrews

**Keywords:** pointing, informativity, infant, pragmatics, communication

## Abstract

Pragmatic theories assume that during communicative exchanges humans strive to be optimally informative and spontaneously adjust their communicative signals to satisfy their addressee’s inferred epistemic needs. For instance, when necessary, adults flexibly and appropriately modify their communicative gestures to provide their partner the relevant information she lacks about the situation. To investigate this ability in infants, we designed a cooperative task in which 18-month-olds were asked to point at the target object they wanted to receive. In Experiment 1, we found that when their desired object was placed behind a distractor object, infants appropriately modified their prototypical pointing to avoid mistakenly indicating the distractor to their partner. When the objects were covered, and their cooperative partner had no information (Experiment 2) or incorrect information (Experiment 3) about the target’s location – as opposed to being knowledgeable about it – infants pointed differentially more often at the target and employed modified pointing gestures more frequently as a function of the amount of relevant information that their partner needed to retrieve their desired object from its correct location. These findings demonstrate that when responding to a verbal request in a cooperative task 18-month-old infants can take into account their communicative partner’s epistemic states and when necessary provide her with the relevant information she lacks through sufficiently informative deictic gestures. Our results indicate that infants possess an early emerging, species-unique cognitive adaptation specialized for communicative mindreading and pragmatic inferential communication which enable the efficient exchange of relevant information between communicating social partners in cooperative contexts.

## INTRODUCTION

Humans are social agents who regularly engage in sophisticated interactions involving cooperation and communication. These interactions require an ability to infer and ascribe goals, intentions, and epistemic mental states – such as knowledge or beliefs – to each other (Frith & Frith, 1999; Grice, 1991; Sperber & Wilson, 1986; Wilson & Sperber, 2012). When interpreting others’ actions humans rely on the assumption that agents act in accordance with their epistemic mental states, even when they represent relevant aspects of reality incorrectly (Dennett, 1978). Epistemic mental states can be induced perceptually, for instance, through gaining visual access to relevant changes in the environment (e.g., Baillargeon et al., 2010; Onishi & Baillargeon, 2005; Surian et al., 2007). Importantly, however, they can also be acquired communicatively from exchanging relevant information between social partners (Sperber, 1997).

Communication can convey various types of information not accessible by direct perception. This includes generic facts about kinds (e.g., koalas eat eucalyptus; Csibra & Gergely, 2009, 2011), information about object referents that are currently absent (e.g., by naming them; Ganea & Saylor, 2013), or causally opaque knowledge about artefact functions or social norms (Gergely & Csibra, 2006). Recent evidence shows that even preverbal infants can understand the communicative function of speech. They expect that through uttering unfamiliar words agents can convey relevant information to their social partners and induce an intended response in them (Martin et al., 2012). Moreover, they can recognize the specific conditions under which an addressee’s epistemic mental state can be modified through communication (Neff & Martin, 2023). For instance, when they observe that an agent – who is unable to retrieve her preferred object – talks to a social partner, 6-month-olds expect the addressee to give the preferred object to the requesting agent. No such expectation is induced, however, when the agent produces involuntary, non-speech sounds such as coughing (Vouloumanos et al., 2014, see also Vouloumanos et al., 2012). These findings suggest that preverbal infants comprehend that through communicative actions an agent can convey information to change the epistemic mental states of their social partner in order to influence her behavior in an intended manner.

Young infants can also infer that interacting social agents can employ communicative actions to update and correct their addressee’s previously formed, but outdated beliefs (Jin et al., 2019; Schulze & Buttelmann, 2022; Song et al., 2008). For instance, 13-month-old infants (Tauzin & Gergely, 2018) could infer that through the turn-taking exchange of partially predictable, unfamiliar signal sequences (Tauzin & Gergely, 2019, 2021) a knowledgeable agent can convey new information about a goal-relevant change to an addressee who has not witnessed it. This result indicates that infants can carry out context-based pragmatic inferences to figure out the intended meaning conveyed by the communicator’s use of novel signals. Moreover, it suggests that infants expect that the relevant information transmitted will modify the addressee’s outdated belief representation to be in line with current reality.

Children not only *comprehend* that communicative signals can induce relevant changes in the epistemic mental states of interactive partners, but they can also *produce* communicative signals in order to elicit such an epistemic change in others (Isaacs & Clark, 1987). In communicative situations where the intended referent of a pointing gesture could be ambiguous for the addressee (O’Neill & Topolovec, 2001) or the addressee lacks relevant information about the communicative context (O’Neill, 1996) 2.5-year-olds repeatedly produce vocal and deictic signals to disambiguate their intended referent. Further studies revealed that infants also produce more points towards a target object in a two-alternative object choice task when the addressee holds an incorrect as opposed to a correct belief about the location of a target object (Knudsen & Liszkowski, 2012a, 2012b).

Such persistence in signaling, however, may simply indicate repeated attempts to elicit an expected instrumental response in others as a means to achieve an intended goal (Townsend et al., 2017). This lean interpretation is also supported by the results of previous comparative studies. As long as their goal remains unfulfilled pet dogs (Gaunet, 2010; Gaunet & Massioui, 2014) as well as non-human great apes (Leavens et al., 2005; Roberts et al., 2014) show persistence in signaling. They repeatedly produce a set of communicative behaviors in their repertoire until they succeed in getting their partner perform the instrumental action needed to satisfy their goal. This suggests that persistence in signaling is an evolutionarily ancient adaptation to gain access to a desired goal through inducing another agent’s means action. This behavioral strategy, however, does not require understanding the social partner’s epistemic mental states.

Recent theories in pragmatics (Grice, 1991; Sperber & Wilson, 1986) argue that a unique characteristic of human communication is that interlocutors spontaneously produce optimally informative communicative signals to provide their interactive partners with relevant information. It is proposed that in order to satisfy the epistemic needs of an addressee communicators infer and rely on the addressee’s mental states of informedness. Based on this, they can generate appropriately informative communicative signals to provide the necessary amount of relevant information that their addressee lacks about the situation (Clark, 2015).

For instance, imagine a mechanic who doesn’t recall the precise location of a tool he is searching for and asks his colleague about it. If it is shared knowledge between them that the tool is in a drawer of a certain cabinet, it is sufficient if the colleague replies with a simple reminder which requires minimal effort to produce (e.g., “*it’s in the drawer”*). However, when no sufficient common ground is shared, the appropriate answer will need to be more elaborate to be sufficiently informative and require more effort to produce (e.g., “*it’s in the top drawer of the cabinet in the corner*”). This indicates that the communicator is sensitive to the relevant epistemic state of informedness of the inquiring partner and provides more detailed information when it is needed. Since a response without specific details would be referentially ambiguous for the inquiring partner a pragmatically sensitive communicator spontaneously adds more relevant information to sufficiently disambiguate his answer.

Thus, in human communication the amount of information the addressee provides tends to be spontaneously tailored to meet the inferred epistemic needs of the addressee. This characteristic is fundamentally different from persistence in signaling. Rather than being induced to react by the addressee’s failure to respond in the required manner (e.g., by handing over the requested object), it depends on the communicator’s evaluation of the requirements of the situational and epistemic context for producing an appropriate response.

It is still an open question, however, whether human infants also possess the ability to adequately adjust their communicative actions as a function of the epistemic state of informedness of their communicative partner. Evidence for this would need to indicate that infants can flexibly modify their communicative actions in order to provide the relevant information necessary to satisfy their communicative partner’s epistemic needs (Sperber & Wilson, 1986). We argue, therefore, that to show such a precocious competence in infants it is necessary to demonstrate that (a) the spontaneous modification, (b) the amount of extra effort invested, and (c) the increased level of required informational content of the communicative response produced vary appropriately with the relevant epistemic mental state of informedness of the communicative partner.

A recent study tested the ability of non-human great apes to adjust their pointing gesture to the situational and epistemic context (Tauzin et al., 2020). In one experiment, great apes were presented with high- and low-quality food items which were placed along a straight line between the human experimenter and the ape participant. In order to obtain their preferred food item from the experimenter, the great apes had to indicate its location by pointing at it. In one condition the low-quality food item was closer to them while the desired high-quality piece of food was placed behind it. Therefore, the apes needed to modify their potentially ambiguous proximal pointing gesture to ensure that they avoid mistakenly indicating for the human partner the low-quality food item at the closer location. In contrast, when the high-quality food item was closer to them, they did not need to modify their proximal default pointing gesture. It was found that the apes produced significantly more modified points when the high-quality food item was distally located behind the low-quality piece of food. This suggests that great apes can adjust their deictic gestures in relation to the spatial arrangement of referential targets in the given situational context.

In the same study, it was also examined whether non-human great apes could take into account the relevant epistemic mental state of the human experimenter when producing their pointing gestures. The researchers used the same setup but hid the food items under opaque cups. Apes had to point either for a knowledgeable experimenter who had seen the hiding of the food items or to an ignorant one who had been absent during the hiding event and did not know where the food items were located. It was hypothesized that in order to provide the ignorant experimenter with the relevant information she lacked apes should produce more appropriately modified pointing gestures when requesting their preferred food which was located behind the low-quality food item. However, there was no significant difference between the ignorant and knowledgeable conditions in the number of modified points that apes generated. This indicates that great apes do not modify their pointing gestures for informative purposes and cannot take into account the relevant epistemic mental states of their addressee when producing communicative signals to request an object.

In the present experiments we used a similar paradigm to test 18-month-old infants. We aimed to investigate whether human infants can (a) exert more effort to (b) spontaneously modify their standard pointing gesture so as to (c) provide relevant information for their communicative partner when the situational context or the relevant epistemic state of informedness of their addressee requires it.

## EXPERIMENT 1

In Experiment 1 (*N* = 24) we investigated whether 18-month-olds can modify their prototypical pointing response to efficiently indicate a target object in a situational context where producing an unmodified pointing gesture could be referentially ambiguous for the addressee. In the familiarization phase, the infant sat at a table facing an experimenter (the future addressee) and two laterally placed objects (the Target and a Distractor) in between them. The experimenter first demonstrated the function of the Target object twice showing that when inserted into a tube music and flashing lights ensued. Then, she placed the objects on a direct line leading from the infant to the experimenter so that one of the objects was closer to the infant, while the other was located further away behind the first object. Subsequently, she verbally requested the infant to show “*where it is*” without naming either of the toys. Following the infant’s first pointing gesture towards the two objects, the experimenter put the indicated object into the testing tube. She expressed happiness when the infant pointed at the Target object which induced the sound and light effects. She showed mild sadness when the Distractor object was indicated which could not induce any effects.

Each infant was tested in three conditions. In the Proximal Target (PT) condition the Target toy was placed at the location closer to the infant while the Distractor occupied the location further away, thus from the infant’s perspective it was behind the Target. In the Distal Target (DT) condition the Target object was placed at the more distal location while the Distractor was located closer to the infant and so from the infant’s perspective it appeared in front of the distal Target. In the Target Alone (TA) condition, there was no Distractor object present and the Target occupied the more distant location from the infant.

We hypothesized that in a communicative context 18-month-olds would aim to be pragmatically relevant and informative when pointing at the Target object they desired to obtain. However, given the spatial arrangement of the Target and the Distractor object in the Distal Target condition, using an unmodified, prototypical pointing gesture could be ambiguous for the addressee as it could be mistakenly interpreted to indicate the Distractor object. We predicted, therefore, that in the Distal Target in contrast to the Proximal Target and Target Alone conditions infants would spontaneously exert more effort to produce modified points and provide their communicative partner with an optimally informative deictic gesture.

### Methods

#### Participants.

Twenty-four infants participated in Experiment 1 (*N* = 24, 12 females) based on an a priori power analysis to reach 0.8 power with medium effect size (*d* ≈ 0.5) in paired-samples *t*-tests to analyze the hypothesized difference between DT vs. PT and DT vs. TA conditions. The mean age of infants was 556 days (*SD* = 13.88). Twelve additional participants were excluded from the analysis of Experiment 1, due to pointing in fewer than four test trials (*N* = 10), repeated parental intervention (*N* = 1) and fussiness (*N* = 1). The recruitment and experimental procedure were approved by the United Ethical Review Committee for Research in Psychology of Hungary.

#### Apparatus.

Infants sat in a highchair (seat height: 54 centimeters) approximately 36 centimeters away from the front edge of the table (height: 71 centimeters, width: 80 centimeters, depth: 70 centimeters) to ensure that they were unable to reach the objects that were placed on the table in the familiarization and test phases. The experimenter sat at the opposite side of the table facing the infant, while the parent sat slightly behind the infant at her right side. If the infant did not point because she appeared to want to sit closer to the parent, it was allowed for the parent to make the infant sit in her lap from the subsequent trial. In this case the highchair was removed, however, the position and distance of the infant from the objects remained the same as in the trials when she sat in the highchair. There were three objects on the table: a plastic tube (height: 18.5 centimeters, size of bottom part: 16 × 13 centimeters), which could emit sounds and light flashes when the Target toy was inserted in it; the Target object, a capsule-shaped plastic toy (height: 6 centimeters, diameter: 4 centimeters); the Distractor object, a plastic cube (height/width/depth: 5 centimeters), which did not fit into the tube, therefore, it could not induce any effects. The toys were placed at predetermined locations during the familiarization and test trials. In the familiarization trials the toys were placed next to each other with 65 centimeters distance in between them and equally distant from the lateral edges of the table. During the test trials the toys were placed at 30 centimeters distance from each other arranged in a row at the mid-line of the table so that one of them was closer to the infant while the other was closer to the experimenter at the opposite side of the table. The toy on the infant’s side was 35 centimeters away from the edge of the table. The tube was constantly present at the same position on the right-hand side of the experimenter close to the corner of the table.

#### Procedure.

After the participant and the parent were seated at their side of the table, the experimenter took her seat at the opposite side facing the infant. The Target and Distractor objects were in the lap of the experimenter, from where she placed them on the table next to each other. Then during both of the two identical familiarization trials the experimenter first looked and smiled at the infant and using infant-directed speech intonation she said the Hungarian equivalent of “*Look at this*!” (“*Figyelj*!”). The experimenter then took the Target object and dropped it into the tube which was activated and played a short melody while producing flashes of light for approximately 2 seconds. The experimenter commented on this effect in a cheerful tone: “*How nice!*” (“*De jó!*”). The lack of affordance of the Distractor object to induce such an effect was not demonstrated during the familiarization.

During the subsequent 12 test trials the experimenter first always looked at the infant and said: “*Look at this!*” using infant-directed speech intonation. She then placed the first object on the table to its pre-specified location which was closer to her. This allowed the infant to have an unobstructed view of the object. Subsequently the experimenter placed the second object on the other location between the infant and the experimenter so that it occupied the position closer to the infant. Finally, the experimenter looked at the participant and asked: “*Where is it? Show it to me!*” (“*Hol van? Mutasd meg!*”) in an ostensive manner. When the experimenter’s request induced a pointing response by the infant, she took the object that the infant’s gesture indicated. If this was the Target object the experimenter grabbed it and dropped it into the tube, which resulted in light and sound effects. Commenting on the induced effect the experimenter said: “*How nice!*” in a cheerful manner. If the infant first pointed at the Distractor object, the experimenter took it and tried to drop it into the tube. Since the Distractor object did not fit into the tube no effects were produced. The experimenter then commented in a sad tone of voice: “*This is no good*,” (“*Ez nem jó.*”). If following the experimenter’s request, the infant did not point for approximately 10 seconds the experimenter repeated her question (“*Where is it? Show it to me!*”). If the participant did not point even after the experimenter’s second request for an additional 10 seconds, the experimenter repeated her request one more time. The trial ended if the infant pointed or if she did not point for approximately 30 seconds. In this case the experimenter said: “*Look at this!*” and demonstrated the function of the Target toy once again.

The test phase involved 4 Distal Target, 4 Proximal Target and 4 Target Alone trials in a pseudorandomized order for each infant. In the Distal Target trials the Target object was located further away from the infant and behind the Distractor object from the infant’s perspective. In the Proximal target trials the Target object was closer to the participant and the Distractor object was placed behind it. In the Target Alone trials, the Target object was placed at the same location as in the Distal Target condition, but this time no Distractor object was placed in between the Target object and the infant.

#### Coding

##### Definition of Pointing.

The responses of the subjects were video recorded from three different angles for offline analysis. Video cameras were positioned in front of and on the two sides of the participant. We defined pointing as a hand action with the palm facing down with a maximum rotation of 90 degrees to the sides and with a protruded finger or fingers (see Kovács et al., 2014) towards the vertical plane determined by the location of the Target and Distractor objects and delimited by the tabletop. Pointing towards the ceiling, the floor or the lateral walls were excluded from the analysis.

##### Definition of Modified Pointing.

We assumed that in the present two-alternative forced-choice task an intentionally modified deictic gesture to inform the addressee about the correct location of the Target object will (a) require extra effort to produce, (b) must be directed towards the vertical plane determined by the position of the Distractor and Target objects delimited by the tabletop and (c) must avoid falling in line with the Distractor.

**Figure 1.**
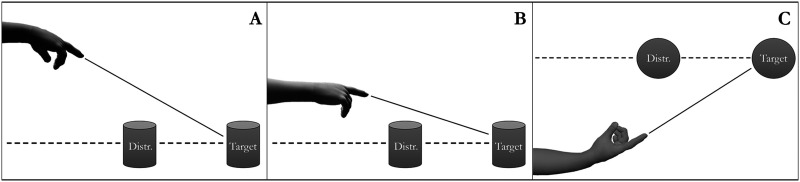
Computer generated imagery of infants’ modified points. Dashed lines show the vector of unmodified points which may intend to indicate the distal Target object but because the extended line of the point first passes through the position of the proximal Distractor, they appear to indicate the Distractor (instead of the Target). Straight lines show the extended vector of modified points indicating the Target referent (while avoiding the Distractor). (A): Type I modified point (shown from the side). (B): Type II modified point (shown from the side). (C): Type III modified point (shown from above).

Based on these assumptions and previous results three different types of modified points could be differentiated. Type I modified point (pointing from above): Infants raised their arm high while lowering their hand so that the extended line of their pointing hand passed significantly above the Distractor object while crossing the Target (Figure 1A). Type II modified point (pointing straight with raised arm): Infants raised their arm so that the extended line of the arm-hand axis was approximately straight and passed sufficiently above the Distractor object while crossing the position of the Target (Figure 1B, see also Gonseth et al., 2017; Roberts et al., 2014). Type III modified point (pointing from the side): Infants moved their arm laterally to the side while rotating their wrist in an angle to point towards the target in a way that the finger(s)’ extended direction would not pass close to the proximal location of the Distractor object while directly crossing the position of the distal Target (Figure 1C, see also Kovács et al., 2014).

#### Data Analysis.

We excluded infants if they were fussy (e.g., crying) or pointing fewer than four test trials in at least two different conditions. The trials in which the parent intervened (pointed towards the objects or verbally named the target) were excluded. If such parental intervention repeatedly occurred and so the infant produced less than four valid trials that could be included in the data analysis, we excluded the infant due to repeated parental intervention.

The main measure of interest was the number of modified points produced to indicate the distal object. We calculated the sum of modified pointing gestures for each individual in each condition separately. We analyzed the data in SPSS 20 using Generalized Estimating Equations (GEE) tests (Hardin & Hilbe, 2002) since GEE is a robust semi-parametric alternative to ANOVA for paradigms relying on a within-subject design. We tested the main effect of Target Location (Distal Target, Proximal Target, Target Alone) to predict modified points as a binary logistic variable. All tests were two-tailed.

All trials were coded by a second coder who was blind to the hypotheses of the experiment. The second coder received videos of individual pointing actions which did not contain the reaction of the experimenter. The coder’s task was to decide whether the pointing was modified or not. Inter-rater reliability was almost perfect (*Kappa* = 0.958).

### Results

**Figure 2.**
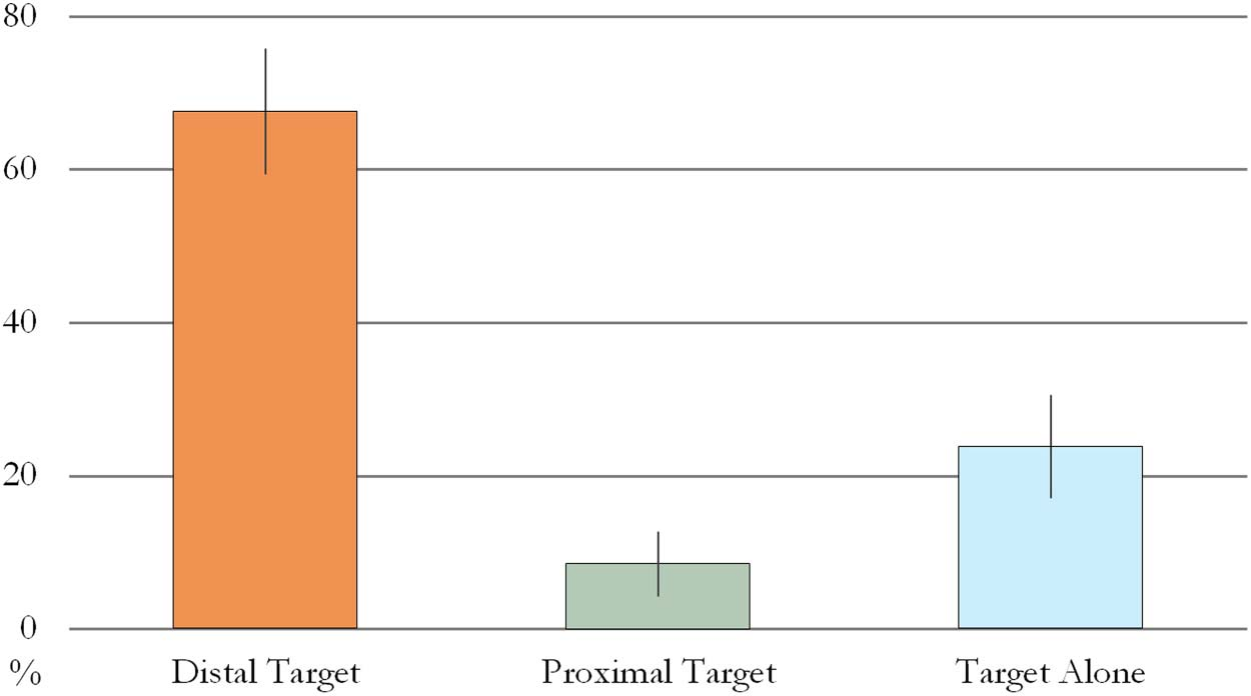
Proportion of modified points in Experiment 1. Error bars represent unpooled SEM.

The GEE analysis revealed that the number of modified pointing gestures significantly differed across the three conditions (Wald *χ*^2^ = 24.841, *p* < 0.001). Infants produced significantly more modified points in the DT (*M* = 67.65%, *SE* = 8.14) than in the PT (*M* = 8.51%, *SE* = 4.11, *p*_Bonferroni_ < 0.001) and TA (*M* = 23.81, *SE* = 6.65, *p*_Bonferroni_ < 0.001) conditions, which allowed them to indicate the Target object when a prototypical unmodified point could have been misinterpreted. We did not find a significant difference between the PT and TA conditions (*p*_Bonferroni_ = 0.096; Figure 2).

### Discussion

The results of Experiment 1 suggest that 18-month-olds can spontaneously modify their deictic gestures depending on the relative location of the Target object and the Distractor. Since in the Distal Target condition the intended referent of a prototypical pointing gesture could have been ambiguous for the addressee, infants adjusted their pointing responses to avoid mistakenly indicating the distractor object. Therefore, they produced more modified points in the Distal Target as opposed to the Proximal Target and Target Alone conditions. This suggests that 18-month-olds selectively modify their pointing gestures for informative purposes when they need to unambiguously indicate their intended referent.

In non-human great apes comparable results were found using a similar paradigm (Tauzin et al., 2020), however, with one crucial difference. Apes also produced significantly more modified points in the Distal Target than in the Proximal Target condition but – in contrast to humans – there was no significant difference in the number of modified points between the Distal Target and Target Alone conditions. In the Target Alone condition, no Distractor object was present while the Target object was placed at the same distal position from the participant as was the Target in the Distal Target condition. The lack of significant difference between these conditions suggests that in apes the production of modified points may only depend on the absolute distance of the Target object from the pointer (see Gonseth et al., 2017). In contrast, human infants spontaneously make an effort to produce disambiguated points *for* their addressee when the given situational context requires it.

Importantly, in Experiment 1 the experimenter had direct visual access to the two objects throughout the procedure. Therefore, it is an open question whether infants could also produce sufficiently informative deictic gestures to provide relevant information that is unavailable to their social partner. This would require them to infer and rely on the relevant epistemic mental state of informedness of their communicative partner to identify her epistemic needs. To investigate this, in Experiment 2 we varied whether the experimenter was informed or uninformed about the location of the Target when the infant produced her pointing gesture to request it from her.

## EXPERIMENT 2

In Experiment 2 (*N* = 48) we examined whether infants can adjust their pointing gestures to provide an ignorant – as opposed to a knowledgeable – communicative partner with the relevant information she lacks to correctly identify the location of the target referent. Experiment 2 was similar to Experiment 1 except that two experimenters were involved in the procedure and the two objects were hidden under opaque cups. The Target object was a self-propelled, wind-up toy that could jump, while the Distractor was an inert building block. Each infant participated in two conditions. In the Correct Information (CI) condition only one experimenter was present who placed the two objects at their respective locations and covered them with two identical opaque cups before leaving the room. When she returned, she asked the infant to show her “where it is”, without naming the Target object. The No Information (NI) condition was identical except that after the experimenter who placed and covered the objects left, it was another experimenter who entered the testing room and asked the infant to show her where the desired Target object was. Since this second experimenter had not witnessed the placement and hiding of the objects, she lacked the relevant information about the location of the Target referent. Therefore, we hypothesized that 18-month-olds would be more likely to (a) point at the Target object and (b) use more often a modified point to do so when the Target was located behind the Distractor in the No Information than in the Correct Information condition.

### Methods

#### Participants.

Forty-eight infants participated in Experiment 2 (*N* = 48, 23 females) based on the recommendations of Brysbaert (Brysbaert, 2019) to reach 0.8 power with medium effect size (*d* ≈ 0.4). The mean age of infants was 556 days (*SD* = 13.12). Twenty-one additional participants were excluded, due to pointing in fewer than four test trials (*N* = 18), repeated parental intervention (*N* = 2) or fussiness (*N* = 1).

#### Apparatus.

The sound emitting tube was not used in Experiment 2 as in Experiment 1 we found that it induced infants to point at it. The Target object was a self-propelled clown toy, which could jump forward after it was wound-up (height: 6.5 centimeters). The Distractor object was the same as in Experiment 1. The Target and Distractor objects were held by Experimenter 1 in her lap, from where she placed them at their respective locations on the table that were pre-specified before the trials. Two identical opaque plastic cups (height: 9 centimeters, diameter at top: 8 centimeters) were used to cover the Target and Distractor objects after they had been placed on the table.

#### Procedure.

In the two trials of the familiarization phase Experimenter 1 and Experimenter 2 were both present and sat in front of the infant on the opposite side of the table. Experimenter 1 was directly facing the infant, while Experimenter 2 sat next to Experimenter 1. First, Experimenter 1 looked and smiled at the participant and addressed her in an ostensive manner: “*Hi, [baby’s name], watch this!*” (“*Szia [baby’s name], nézd csak!*”). She then took the Target object from her lap, placed it on the table and demonstrated its functional use to the infant by winding it up to make it to jump on the table in a self-propelled manner. Experimenter 1 commented on the Target toy’s jumping action in a cheerful tone, saying: “*How nice!*”. Then she told the infant: “*Look!*” (“*Nézd!*”) and placed first the Target toy then the Distractor object on either side of the table. Experimenter 1 then covered the two objects by putting identical opaque cups over them in full view of the infant. If in the meantime the infant looked away, Experimenter 1 stopped and gently knocked on the table with the opaque cup until the infant looked back, and only then did she continue putting the cups over the objects. When she finished, she made eye-contact with the participant again and asked her in an ostensive manner: “*Where is it? Show it to me!”*.

When the experimenter’s request induced a pointing response, she reached out for the object that the infant’s pointing indicated. If this was the Target object the experimenter took it, wound it up and made it jump on the table in a self-propelled manner. While watching the Target object jump she commented “*How nice!*” in a cheerful manner. If the infant pointed at the Distractor object, the experimenter took that object from under the cup and put it down on the table. Looking at the inert Distractor object in front of her she commented in a sad tone of voice: “*This is no good*.” (“*Ez nem jó.*”). If the infant did not point for approximately 10 seconds, the experimenter repeated her request. If the participant failed to point even after the second request for an additional 10 seconds, the experimenter repeated her request one more time. The trial ended if the infant pointed or if she did not point for approximately 30 seconds. In the latter case the experimenter said to the infant: “*Look at this!*” and went on to demonstrate the functional use of the Target toy.

The second familiarization trial was the same as the first except that after the objects were covered up, the two experimenters – while sitting in their chairs – moved sideways in the same direction. As a result, it was now the second experimenter who was directly facing the participant while the first experimenter sat next to the second experimenter. This time it was the second experimenter who asked the infant to show where the Target object was in order to familiarize the participant with the fact that either of the two experimenters can ask them to point at the Target.

Subsequently, before the test trials the experimenter who first placed and covered the objects left the testing room. The objects were placed at their respective positions as in Experiment 1, with the difference that in Experiment 2 they were also covered by opaque cups. The Target object was placed either further away (Distal Target condition) or closer (Proximal Target) to the infant. There were no Target Alone trials in Experiment 2. During the test trials after the experimenter finished covering up the objects, she left the room. In the Correct Information (CI) condition the same experimenter entered the room again after approximately 3 seconds of delay. In the No Information (NI) condition it was the other experimenter – who was not present when the objects were placed and covered up – who entered the room approximately 3 seconds after the first experimenter had left. The experimenter who returned then asked the infant: “*Where is it? Show it to me!*”. She reacted to the infant’s pointing (or lack of response) in the same way as in the familiarization trials of Experiment 2.

Experiment 2 consisted of 12 test trials for each infant: 3 No Information (NI) trials where Experimenter 1 (E1) was the addressee (NI/E1), 3 NI/E2 trials, 3 Correct Information trials (CI/E1) trials, and 3 CI/E2 trials. The trials were presented in mini blocks (e.g., 3 CI/E1 in a row). The order of mini blocks was fully counterbalanced across participants. The location of the Target object was pseudorandomized within participants.

#### Data Analysis.

The main measures of interest were the number of pointing at the target object and the number of modified points in the Correct Information and No Information conditions. We calculated the sum of target pointing and modified pointing gestures for each individual in each condition separately. We analyzed the data using binary logistic GEE tests with main effects of Condition (Correct Information, No Information) and Target Location (Distal Target, Proximal Target). All tests were two-tailed.

All trials were coded by a second coder who was blind to the hypotheses of the experiment. The second coder coded videos of the individual pointing actions which, however, did not contain the preceding placement of the objects or the subsequent reaction of the experimenter. The coder’s task was to decide whether the pointing was modified or not. Inter-rater reliability was almost perfect (*Kappa* = 0.964).

### Results

**Figure 3.**
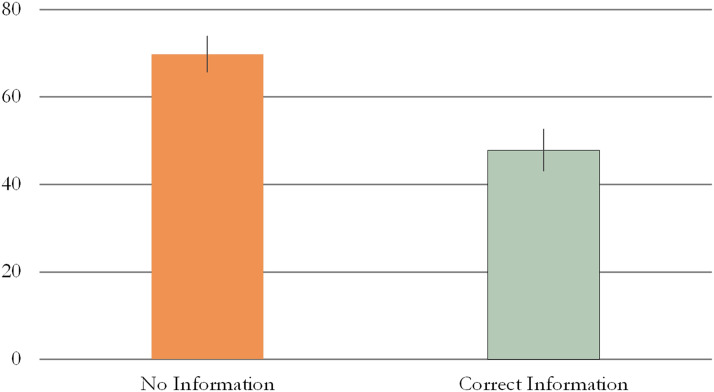
Proportion of pointing at target in Experiment 2. Error bars represent unpooled SEM.

We found that infants pointed significantly more at the target in the NI (*M* = 69.84%, *SE* = 4.15) than in the CI (*M* = 47.87%, *SE* = 4.78) condition (Wald *χ*^2^ = 6.845, *p* = 0.009, Cramer’s *V* = 0.38, see Figure 3).

**Figure 4.**
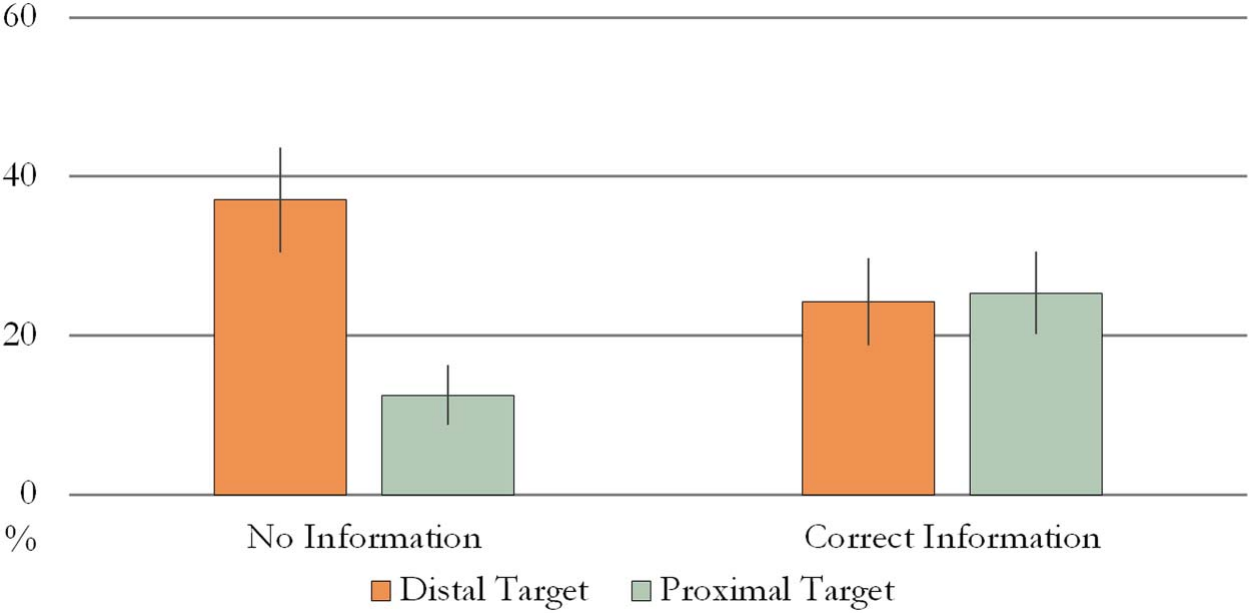
Proportion of modified points in Experiment 2. Error bars represent unpooled SEM.

A further GEE analysis with the number of modified points as the dependent variable revealed a significant Condition × Target Location interaction (Wald *χ*^2^ = 7.44, *p* = 0.006, Cramer’s *V* = 0.39) as 18-month-olds produced more modified points in the NI condition when the target was distally located (NI-DT: *M* = 37.04%, *SE* = 6.63; NI-PT: *M* = 12.5%, *SE* = 3.72; CI-DT: *M* = 24.19%, *SE* = 5.48; CI-PT: *M* = 25.35%, *SE* = 5.2; see Figure 4).

### Discussion

The findings of Experiment 2 suggest that in a communicative context 18-month-olds can attribute epistemic mental states about a relevant fact to their addressee. Having inferred that their communicative partner lacked information about the location of the hidden objects, infants pointed at the target referent more often than they did for a communicative partner who knew where the Target object was located. They also made more effort and more frequently employed modified deictic gestures when it was necessary to provide relevant information to their uninformed partner. These findings indicate that in communicative situations 18-month-olds can rely on their precocious capacity for context-based pragmatic inferences to produce optimally informative communicative actions.

In a previous study (Tauzin et al., 2020) with non-human great apes no evidence was found to indicate that apes would also produce more modified pointing gestures to inform an ignorant – as opposed to a knowledgeable – addressee. Although great apes are able to adjust their deictic gestures to the situational context (Gonseth et al., 2017; Leavens et al., 2005; Tauzin et al., 2020) and they can also keep track of other agents’ state of informedness to predict their behavior (Krupenye & Call, 2019; Krupenye et al., 2016), they seem to be unable to use these abilities to satisfy the epistemic needs of a cooperative partner in a communicative situation.

Thus, the present study demonstrates that, in contrast to apes human infants can rely on their communicative partner’s mental state of informedness to present her with sufficiently informative communicative gestures. Experiment 2, however, still left open the question whether apart from recognizing when their communicative partner is uninformed, infants are also sensitive to when their addressee has incorrect information about a relevant fact. Therefore, we conducted a further study to examine this issue.

## EXPERIMENT 3

In Experiment 3 (*N* = 48) we investigated whether infants would produce sufficiently informative pointing gestures to correct their communicative partner’s outdated information about the current location of a target object. Infants were tested in two conditions in a similar setup as in Experiment 2. In the Correct Information (CI) condition the first experimenter placed the Target and Distractor objects at their respective locations and covered them with two identical looking cups. Then, in full view of the first experimenter, the second experimenter removed the two objects from under the cups, swapped them, and covered them again. Subsequently, the first experimenter left the room. When she returned again, she asked the infant to show the location of the target object. The Incorrect Information (II) condition was the same except that having placed and covered up the two objects, the first experimenter immediately left the room. As she could not see that during her absence the two objects had been swapped by the second experimenter, she had outdated, incorrect information about the location of the Target object when she returned. We hypothesized that if infants could recognize that their communicative partner possessed incorrect information about the location of the Target object and were motivated to correct it, they would be more likely to (a) point at the Target in the Incorrect Information than in the Correct Information condition and (b) do so more often by using a modified pointing gesture when the Target is distally located behind a distractor object.

We further explored if there is a difference between the Incorrect Information condition of Experiment 3 and the No Information condition of Experiment 2. Having incorrect information about the location of the requested Target object would always lead the experimenter to choose the wrong container. In contrast, being uninformed about the Target’s location would result in guessing and finding the target by chance in half of the trials. We, therefore, employed further exploratory analysis to investigate whether infants provide more disambiguated, relevant information to their partner who has incorrect – as opposed to no – information about the Target’s location to increase the likelihood of retrieving it.

### Methods

#### Participants.

Forty-eight infants participated in Experiment 2 (*N* = 48, 22 females) based on the calculations of Brysbaert (2019) to reach 0.8 power with medium effect size (*d* ≈ 0.4). The mean age of infants was 557 days (*SD* = 11.68). Twenty-eight additional participants were excluded, due to pointing in fewer than four test trials (*N* = 27) and repeated parental intervention (*N* = 1).

#### Apparatus.

The setup was the same as in Experiment 2.

#### Procedure.

The two familiarization trials of Experiment 3 were similar to those of Experiment 2 except that after having placed the opaque cups on the two objects – which was witnessed by both experimenters as well as the participant – the first experimenter said: “*I’m going out for a while, but I’ll be right back*” (“*Most kimegyek egy kicsit, de mindjárt visszajövök*”). The other experimenter followed her with her eyes until she left the room, then turned away from the infant and knocked softly on the wall once to indicate for the first experimenter in the other room that she can come back now. After approximately 3 seconds delay the first experimenter returned and asked the infant: “*Where is it? Show it to me!*”. She reacted to the infant’s points in the same way as in Experiment 2. In the second familiarization trial, the roles of the two experimenters were reversed.

Before the test trials both experimenters were present in the testing room. In the Incorrect Information (II) condition the experimenter who first placed and covered up the objects announced “*I’m going out for a while, but I’ll be right back*” and then left the room. Then the other experimenter raised the opaque cups and revealed the Target and Distractor objects to the infant. She swapped the two objects and covered them again with the cups. If the infant looked away, the experimenter waited until the infant looked back and then finished covering up the objects. Afterwards the experimenter turned away and softly knocked on the wall once to indicate to the first experimenter that she can return. The first experimenter came back, sat down and asked the infant “*Where is it? Show it to me!*”. She reacted to the infant’s pointing response in the same way as in Experiment 2. The procedure of the Correct Information (CI) condition was exactly the same except that the experimenter left the room only after she had witnessed the second experimenter swapping and covering up the Target and Distractor objects. Therefore, when she returned she was knowledgeable about final position of the two objects.

Experiment 3 consisted of 8 test trials due to the increased duration of them: 2 II/E1 trials (where the first experimenter returned with an incorrect information about the location of the Target and Distractor objects), 2 II/E2 trials, 2 CI/E1 trials (where the first experimenter returned with correct information about the location of the Target and Distractor objects) and 2 CI/E2 trials. The trials were presented in mini blocks (e.g., two II/E1 trials in a row). The order of mini blocks was fully counterbalanced across participants. The location of the Target object was pseudorandomized within participants.

#### Data Analysis.

We analyzed the data using binary logistic GEE tests with main effects of Condition (Incorrect Information, Correct Information) and Target Location (Distal Target, Proximal Target). We conducted a further Generalized linear mixed model (GLMM) analysis with pointing at target as the predicted binary variable. The model included the fixed effects of Condition (Incorrect Information, No Information), Target Location (Distal Target, Proximal Target) and Subject as a random effect. GLMM as a semi-parametric alternative to ANOVA was used instead of GEE to analyze between-subject contrasts, as GEE is only suitable to test within-subject differences. All tests were two-tailed.

All trials were coded by a second coder who was blind to the hypotheses of the experiment. The second coder received videos of individual pointing actions that did not include the placement of the objects and the reaction of the experimenter. The coder’s task was to decide whether the pointing was modified or not. Inter-rater reliability was almost perfect (*Kappa* = 0.987).

### Results

**Figure 5.**
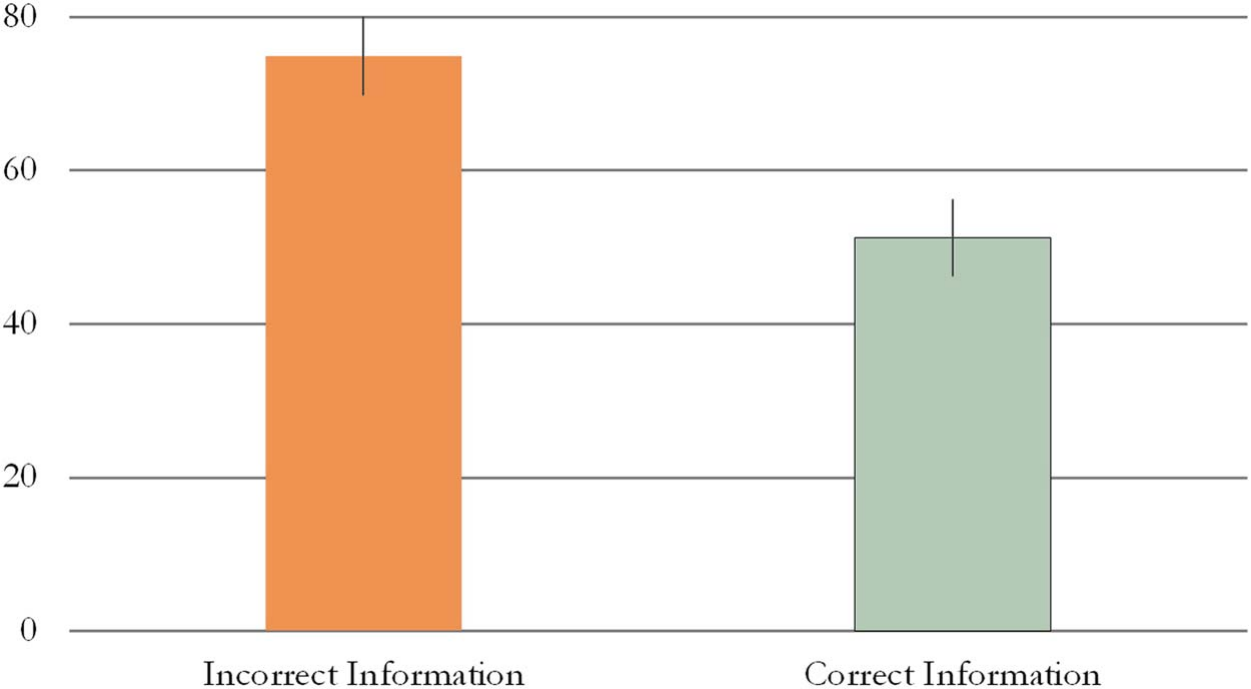
Proportion of pointing at target in Experiment 3. Error bars represent unpooled SEM.

Infants produced more points at the target in the II (*M* = 75%, *SE* = 5.04) than in CI (*M* = 51.25%, *SE* = 5.01) condition of Experiment 3 (Wald *χ*^2^ = 8.336, *p* = 0.004, Cramer’s *V* = 0.42; see Figure 5).

**Figure 6.**
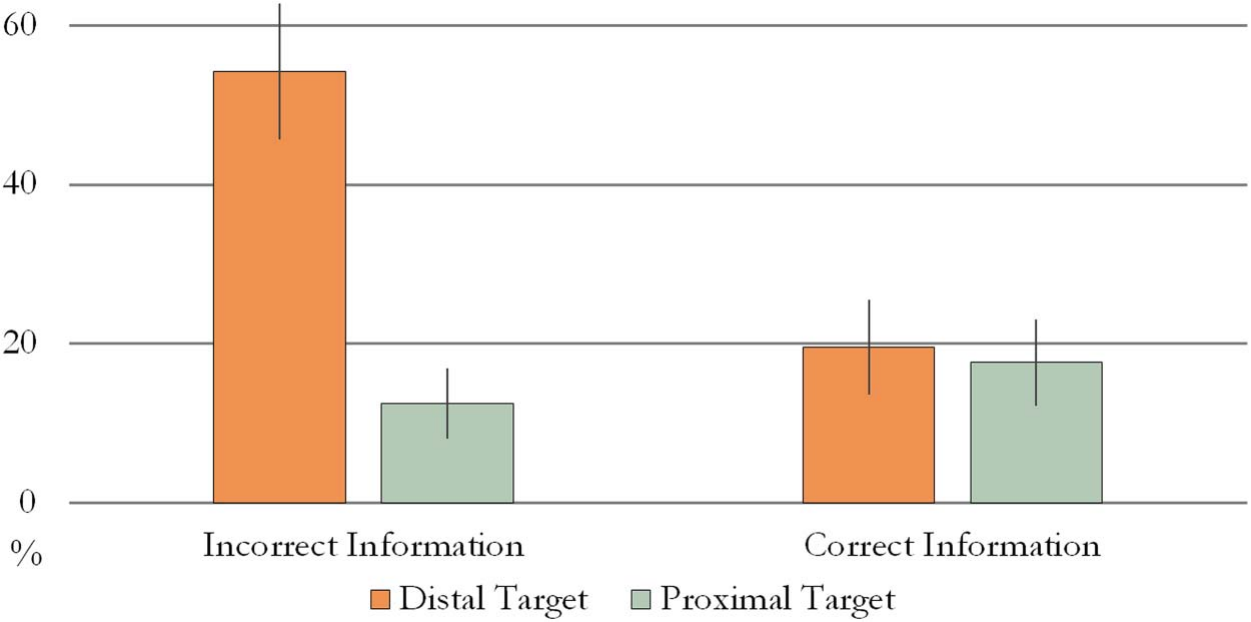
Proportion of modified points in Experiment 3. Error bars represent unpooled SEM.

There was a significant Condition × Target Location interaction (Wald *χ*^2^ = 7.419, *p* = 0.006, Cramer’s *V* = 0.39) when the dependent variable was the number of modified points (II-DT: *M* = 54.29%, *SE* = 8.54; II-PT: *M* = 12.5%, *SE* = 4.46; II-DT: *M* = 19.57%, *SE* = 5.91; II-PT: *M* = 17.65%, *SE* = 5.39; see Figure 6).

We further analyzed if there is a significant difference in the number of points produced between the Incorrect Information condition of Experiment 3 and the No Information condition of Experiment 2. We first analyzed if there was a difference in the number of points produced between these conditions. We found no significant difference, therefore, we could conduct a GLMM analysis to compare the two groups to explore whether infants more often point at the target in the Incorrect Information as opposed to the No Information condition. The GLMM analysis revealed that infants produced significantly more pointing at the target in the Incorrect Information than in the No Information condition (*F*(1, 221) = 4.035, *p* = 0.046, *d* = 0.41).

### Discussion

The findings of Experiment 3 also showed that when interacting with a social agent in a cooperative situation 18-month-olds can infer and rely on their partner’s relevant epistemic mental states. When their interlocutor had incorrect information about a relevant fact, infants made more effort and produced appropriately informative pointing gestures to update their addressee’s outdated mental representation about the current state of affairs.

Our further exploratory analysis showed that 18-month-olds produced significantly more pointing at the Target in the Incorrect Information than in the No Information condition. In a two-alternative object choice task an uninformed social partner could find the Target object in fifty percent of the cases. In contrast, a partner relying on incorrect information about the location of the Target would always fail to find it. Our results, thus, suggests that infants made more effort in the Incorrect Information than in the No Information condition to make sure that their communicative partner would not mistakenly choose the incorrect container. The present findings, therefore, indicate that infants may be able to differentiate between another agent being in an epistemic state of ignorance or having incorrect, outdated information about a relevant fact.

The three experiments, thus, converge to show that in cooperative social contexts 18-month-olds possess pragmatic inferential abilities that they can rely on when engaging in communicative interactions with their social partner. This early pragmatic sensitivity helps them to appropriately adjust their communicative signals to provide the sufficient amount of relevant information that their communicative partner needs to achieve their common goal.

## GENERAL DISCUSSION

During communicative exchanges adult speakers invest mental effort to infer their communicative partners’ epistemic mental states and satisfy their informational needs by providing them with relevant information they lack (Clark, 2015; Grice, 1991; Sperber & Wilson, 1986; Wilson & Sperber, 2012). We found that when the situational and epistemic context makes it necessary even 18-month-olds invest more effort to spontaneously adjust their pointing gestures in order to provide appropriately informative referential signals to their addressee. Previous findings on infants’ communicative pointing (O’Neill, 1996; O’Neill & Topolovec, 2001) could be accounted for by assuming that they only reveal persistent signaling until a partner’s required means action is induced to obtain their goal. This behavioral strategy, however, does not require understanding the other’s epistemic mental states. In contrast, the present results indicate that 18-month-olds can infer and rely on their cooperating partner’s relevant epistemic mental state of informedness. Therefore, they can identify her epistemic needs and are able to flexibly generate appropriately informative deictic signals to provide her the missing information necessary to realize their shared goal.

One may raise the question, however, why the infants in the Correct Information conditions of Experiment 2 and 3 did not produce more informative modified points to specify the location of the Target in the Distal Target than in the Proximal Target condition. This finding also appears unexpected when compared with the results of Experiment 1 where a significant difference was found between these conditions. We conjecture that this pattern of responses may reflect that due to differences in the pragmatic context, the same verbal request of the experimenter received different interpretations by the infants in Experiment 1 than in Experiments 2 and 3.

In Experiment 1, after having placed the objects herself the experimenter never left the room and so she had uninterrupted visual access to the uncovered Target and Distractor objects throughout the procedure. Thus, all the relevant information was available to the experimenter when she asked the infant to show her the location of the Target object. For this reason the infants may have found it unlikely that the knowledgeable experimenter’s question was intended as a genuine request for information she needed. Therefore, the infants may have opted for the pragmatically more plausible interpretation that the experimenter’s communicative intention was to request them to display their communicative ability to correctly indicate the location of their desired object. This required them to produce more modified pointing in the Distal Target condition than in the Proximal Target condition. In contrast, in the Correct Information conditions of Experiment 2 and 3 the experimenter’s direct informational contact with the objects was interrupted when she left the room. Moreover, she had no direct visual access to the objects even when she returned as both objects were covered with identical cups. In this pragmatic context the experimenter’s verbal inquiry could be justifiably interpreted as an *epistemic request* to be informed – if necessary – about possible changes of the relevant state of affairs that may have taken place after she had left the room. However, since no such change took place, there was no need for the infants to update the relevant common ground they already shared with the experimenter. As a result, infants did not invest extra effort to disambiguate their pointing in order to provide the experimenter with new information she didn’t already have to enable her to find the target object. In the given epistemic context it was sufficient to point at their requested target object in a similar manner in the Distal Target and the Proximal Target conditions as in both cases they could assume that the experimenter already shared with them the relevant information about its location. As a result, there was no significant difference in the amount of modified pointing produced to indicate the Distal Target versus the Proximal Target in the Correct Information conditions of Experiments 2 and 3.

Recent studies demonstrated that in non-communicative tasks great apes can rely on the inferred epistemic mental states of others to predict their instrumental, goal-directed behavior (Krupenye & Call, 2019; Krupenye et al., 2016) similarly to human infants (Baillargeon et al., 2010; Onishi & Baillargeon, 2005; Surian et al., 2007). However, it appears that in a communicative context, great apes fail to rely on their mentalization ability. In contrast to human infants (Bohn et al., 2016; Tauzin & Gergely, 2018), they are unable to infer the informative intention of an interactive social partner who uses communicative gestures with a cooperative intent to provide the apes with relevant information (Bohn et al., 2016; Tempelmann et al., 2013). Moreover, in communicative contexts great apes also fail to employ their capacity for referential pointing to produce sufficiently informative deictic gestures to convey relevant information to their cooperative partner. For instance, they do not produce more modified points to inform an ignorant (as opposed to a knowledgeable) communicative partner about the location of a desired piece of food (Tauzin et al., 2020). The empirical evidence, therefore, indicates that in a communicative task young human infants can outperform adult non-human great apes (Moll & Tomasello, 2007).

This difference in performance across species suggests that humans may have evolved a specialized cognitive skillset to support communicative information exchange between cooperating social partners. Thus, humans can rely on their specialized communicative mentalization abilities to achieve two types of epistemic goals. First, they can generate appropriate communicative actions that can change the epistemic mental states of their social partner by providing her with relevant information she lacks. Second, they can infer the intended meaning manifested by their social partner’s communicative actions in the given situational and epistemic context (Bohn et al., 2016; Tauzin & Gergely, 2018). Therefore, we propose that communicative mentalization is a specialized and species-unique cognitive adaptation whose essential function is to support ostensive communication between cooperating social partners (Gergely & Jacob, 2012; Sperber & Wilson, 2002). We argue that it enables efficient and flexible cooperative interactions between humans pursuing a variety of instrumental goals (e.g., in helping, Knudsen & Liszkowski, 2012a, 2012b) as well as playing a crucial role in achieving shared epistemic goals, such as transmitting cultural knowledge to future generations (as in pedagogical teaching contexts, Csibra & Gergely, 2011).

The present study differs in two key aspects from previously employed standard theory of mind paradigms (Dennett, 1978) that became dominantly used to test children’s ability to attribute epistemic mental states to others to predict their future actions (Wellman et al., 2001; Wimmer & Perner, 1983). First, in our experiments infants had to ascribe epistemic mental states to their *communicative partner* instead of a non-interactive protagonist they observed. Second, instead of predicting an instrumental agent’s goal-directed behavior, they had to convey relevant information to change the epistemic mental state of their social partner in a *cooperative situation* to achieve their joint goal. In such an interactive task we found that even 18-month-olds possess early emerging communicative mentalization skills and pragmatic inferential abilities that support cooperation between communicative partners (see also Knudsen & Liszkowski, 2012a, 2012b). This species-unique cognitive system may be a specialized adaptation that enables collaborating social partners to infer and keep track of each other’s relevant epistemic mental states. Furthermore, it may also allow them to exchange appropriately informative communicative signals to *change* each other’s relevant epistemic states to enable them to achieve their instrumental or epistemic goals. Communicative mentalization, thus, differs from the basic theory of mind mechanism whose specialized function is to predict the future actions of instrumental agents. Since the latter ability is present both in humans (Baillargeon et al., 2010; Onishi & Baillargeon, 2005; Surian et al., 2007) and in non-human primates (Krupenye & Call, 2019; Krupenye et al., 2016) the cognitive adaptation to attribute mental states to others to anticipate their instrumental actions might not be a species-specific competence that distinguishes humans from non-human primates. Based on our findings, we propose that the cognitive adaptation for mindreading that is unique to humans is our early emerging capacity for communicative mentalization and our pragmatic inferential skills for ostensive communication. This specialized ability might have evolved to support collaboration and communication between human social partners. Since it is present already in young infants it may be argued to play an essential role in the transmission of social and cultural knowledge across generations.

## ACKNOWLEDGMENTS

We thank M. Bohn, P. Jacob, and D. Sperber for their help with the manuscript. We thank D. Mészégető, P. Kármán, D. Kerschner, T. Sugár, Z. Üllei and J. Horváth for their help in conducting the studies. This research was supported by the European Research Council (ERC) under the European Union’s Seventh Framework Programme (FP7/2007-2013)/ERC Grant 609819 (SOMICS), and the Cognitive Science Hub Seed Grant of the University of Vienna.

## FUNDING INFORMATION

This research was supported by the European Research Council (ERC) under the European Union’s Seventh Framework Programme (FP7/2007-2013)/ERC Grant 609819 to GG and JC and the Vienna CogSciHub Seed Grant to TT.

## AUTHOR CONTRIBUTIONS

TT designed research, analyzed the data; TT, JC, GG wrote the paper.

## DATA AVAILABILITY STATEMENT

Data available at: https://osf.io/gpx27/?view_only=2d12f28e07e344aa9950400196405c73.
